# Genome-Wide Analysis of Hypoxia-Responsive Genes in the Rice Blast Fungus, *Magnaporthe oryzae*

**DOI:** 10.1371/journal.pone.0134939

**Published:** 2015-08-04

**Authors:** Jaehyuk Choi, Hyunjung Chung, Gir-Won Lee, Sun-Ki Koh, Suhn-Kee Chae, Yong-Hwan Lee

**Affiliations:** 1 Division of Life Sciences, College of Life Sciences and Bioengineering, Incheon National University, Incheon 406–772, Korea; 2 Department of Agricultural Biotechnology, Seoul National University, Seoul 151–921, Korea; 3 Fungal Bioinformatics Laboratory, Seoul National University, Seoul 151–921, Korea; 4 Department of Biochemistry, Paichai University, Daejeon 302–735, Korea; 5 Center for Fungal Pathogenesis, Seoul National University, Seoul 151–921, Korea; 6 Center for Fungal Genetic Resources, Seoul National University, Seoul 151–921, Korea; 7 Plant Genomics and Breeding Institute, Seoul National University, Seoul 151–921, Korea; 8 Research Institute of Agriculture and Life Sciences, Seoul National University, Seoul 151–921, Korea; University of Nebraska-Lincoln, UNITED STATES

## Abstract

Rice blast fungus, *Magnaporthe oryzae*, is the most destructive pathogen in the rice-growing area. This fungus has a biotrophic phase early in infection and later switches to a necrotrophic lifestyle. During the biotrophic phase, the fungus competes with its host for nutrients and oxygen. Continuous uptake of oxygen is essential for successful establishment of blast disease of this pathogen. Here, we report transcriptional responses of the fungus to oxygen limitation. Transcriptome analysis using RNA-Seq identified that 1,047 genes were up-regulated in response to hypoxia. Those genes are involved in mycelial development, sterol biosynthesis, and metal ion transport based on hierarchical GO terms, and are well-conserved among three fungal species. In addition, null mutants of two hypoxia-responsive genes were generated and their roles in fungal development and pathogenicity tested. The mutant for the sterol regulatory element-binding protein gene, *MoSRE1*, exhibited increased sensitivity to a hypoxia-mimicking agent, increased conidiation, and delayed invasive growth within host cells, which is suggestive of important roles in fungal development. However, such defects did not cause any significant decrease in disease severity. The other null mutant, for the alcohol dehydrogenase gene *MoADH1*, showed no defect in the hypoxia-mimicking condition (using cobalt chloride) and fungal development. Taken together, this comprehensive transcriptional profiling in response to a hypoxic condition with experimental validations would provide new insights into fungal development and pathogenicity in plant pathogenic fungi.

## Introduction

Oxygen (O_2_) is an essential element for most eukaryotes due to its roles in the maintenance of many physiological processes such as the biosynthesis of heme, sterols, and fatty acids, and also as a terminal electron acceptor in oxidative phosphorylation. Oxygen concentration ranges from 0% to 20.9% in the environment. Habitats lacking oxygen are known as anoxic, while those at the higher end of the oxygen range are known as aerobic [1]. Microbes living in those niches can be exposed to diverse oxygenic environments including hypoxia (a reduced oxygen level associated with physiological and pathophysiological processes) [2], and have adapted to microbial growth in their hosts. The exposure to oxygen depletion; i.e., hypoxia, dramatically influences various physiological processes [3–5] and global transcriptional regulation in eukaryotic cells [6, 7]. Thus, microbes have evolved a set of cellular and molecular mechanisms to survive in low-oxygen environments.

**Fig 1:**
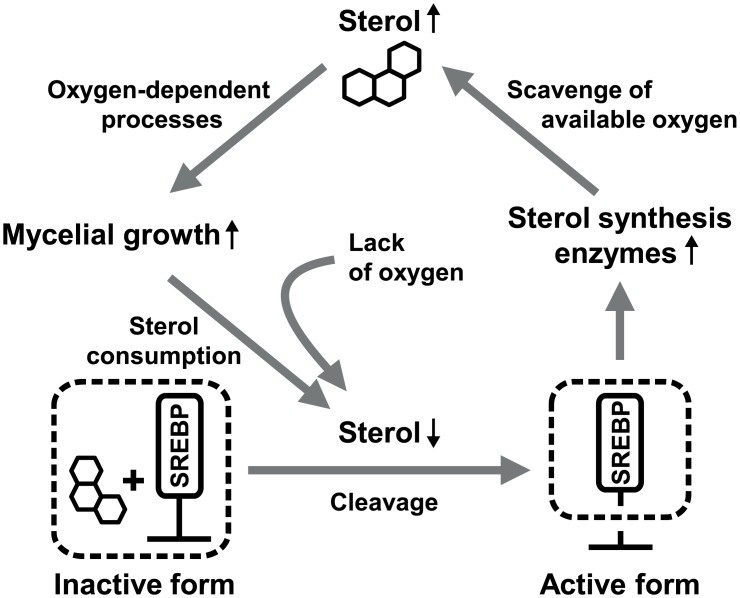
Schematic diagram of SREBP regulation for adaptation to hypoxia. Sterols control activation of SREBP: it is inactive in the presence of and active in the absence of sterols. Growth in limited oxygen conditions inevitably resulted in a lack of sterols, activating SREBP. Increases in enzymes for sterol biosynthesis and other oxygen-dependent pathways produce more sterols, which can be used for mycelial growth.

In healthy humans, oxygen levels vary depending on the location in the body, ranging from 2.5% in the kidneys to 9% in the lungs [1, 8]. Human fungal pathogens, therefore, encounter changes in oxygen concentration and also can cause oxygen depletion (hypoxia) when they invade host tissue. For example, *Aspergillus fumigatus* infects the lungs of mice where it is exposed to the hypoxic microenvironment, and adapts its physiological processes to overcome this. One of the adaptation processes is to change energy metabolism to a mechanism such as ethanol fermentation, resulting in accumulation of ethanol [9]. A null mutant for the alcohol dehydrogenase gene caused increased host inflammatory responses while still causing aspergillosis to the same degree as the wild-type [9]. Another example of fungal response to hypoxia is the sterol regulatory element-binding protein (SREBP) pathway involved in regulation of sterol biosynthesis [10]. As shown in Fig 1, membrane-bound SREBP is activated when sterols are depleted due to hypoxia. Active SREBP turns on the expression of sterol synthesis enzymes and other oxygen-dependent proteins. SREBP is essential for virulence and growth under hypoxic conditions in *Cryptococcus neoformans* and *A*. *fumigatus* [11–13], suggesting that adaptation to hypoxia is important for fungal virulence in mammals. However, little is known about hypoxia responses of plant pathogenic fungi and the importance of such responses in disease establishment within plants.

In plant tissues, active metabolic reactions result in low internal oxygen concentrations even under normal oxygen (normoxia) conditions [14]. For example, a decrease of 1–12% in internal oxygen levels has been reported in petioles, root meristems, phloems, and seeds of various plants [5, 15–17]. However, less has been reported about the internal oxygen concentration in leaves, where oxygen is produced. When infected with biotrophic plant pathogens (obligate parasites), hosts and pathogens must compete for oxygen as well as nutrients. Such competition might lead to decreased availability of oxygen. In particular, in darkness, plants use oxygen through respiration without photosynthesis or production of oxygen, and the internal oxygen level in the leaves decreases. Thus, plant-invading biotrophic agents who encounter oxygen-depleted environments are able to adapt to hypoxic conditions for successful infection.

Rice blast caused by *Magnaporthe oryzae* is a socioeconomically important disease that results in enormous yield loss [18]. The *Oryza sativa*-*M*. *oryzae* pathosystem has been studied as a model system of plant-fungal pathogen interactions. This fungus establishes a biotrophic interaction within the host early in the infection process and then switches to a necrotrophic lifestyle [19]. Dramatic developmental changes occur in the fungus during establishment of infection in the host. Firstly, a dome-shaped appressorium is developed at the tip of a germ tube. The appressorium matures as glycerol accumulates within it, generating high osmotic pressure. [20]. A penetration peg from the mature appressorium pierces the plant cell wall with mechanical force and forms a thin primary hypha down into the plant cell [21]. Secondly, the primary hypha differentiates into thick bulbous infectious hyphae that fill the first-invaded cell, and then moves to neighboring cells. Later in infection, the fungus switches lifestyle to a necrotrophic phase in which it kills neighboring host cells and gains nutrients from the dead cells.

We hypothesized that plant pathogens are exposed to hypoxic microenvironments during a biotrophic phase in host cells and their ability to overcome this challenge is essential for the successful establishment of disease. High-throughput RNA sequencing (RNA-Seq) was employed to analyze transcriptional responses of the fungus to hypoxic environments and to compare our results to those of other fungal pathogens and to *in planta* transcriptomes. Additionally, null mutants for the specific hypoxia-responsive genes were generated and tested for their development and pathogenicity on rice.

## Materials and Methods

### Sampling under hypoxia culture conditions

*Magnaporthe oryzae* strain KJ201 was incubated in liquid complete medium (LCM; 0.6% yeast extract, 1% sucrose, and 0.6% tryptone) at room temperature for three days. Homogenized mycelia were filtered through two layers of cheesecloth. Mycelia were harvested using Miracloth (Calbiochem, CA, USA) after three washes with 0.9% NaCl. The harvested mycelia were resuspended in 20 ml of 0.9% NaCl and 500 μl of the suspension were inoculated on V8 juice agar plates (8% V8 juice and 1.5% agar, pH 6.7) layered with a 0.45-μm pore cellulose nitrate membrane filter (Whatman, Maidstone, England). All 20 plates were pre-incubated for three hours at 25°C. Ten of these plates were then incubated in the hypoxia chamber (Coy Lab Products, MI, USA), at 99% N_2_ and 1% O_2_. The other 10 were incubated outside of the chamber (normoxia control). After 12 hours of incubation, mycelia were harvested from the plates. The harvested fungal tissues were frozen immediately in liquid nitrogen and stored at -80°C for RNA isolation.

### RNA isolation and RNA-Seq

Total RNAs were extracted using TRIzol reagent (Life Technologies, MD, USA). The integrity of RNA was estimated using Bioanalyzer and Pico RNA chip, following the eukaryotic total RNA assay protocol [22]. The TruSeq RNA sample preparation kit (Illumina, CA, USA) was used, following the manufacturer’s instructions. Briefly, poly A tailed transcript RNA was isolated by oligo(dT) selection using streptavidin-coated magnetic beads and fragmented randomly by Mg^2+^ ion treatment. The fragmented mRNA was subjected to cDNA synthesis using Superscript II reverse transcriptase and random hexamers (Invitrogen, CA, USA). RNAs with a length of 300 bp to 400 bp were selected and sequenced on the HiSeq 2000 system (Illumina, CA, USA).

### Read filtering, mapping, and expression quantification

Paired-end reads were filtered as follows: 1) Adaptor sequences were trimmed from the reads using the NGS QC toolkit (ver. 2.3) [23]. 2) Reads with less than 70% of ‘Q > 20’ bases were removed. 3) Only paired reads were selected for further analyses. TopHat (ver. 2.0.8b) [24] was used to map the filtered reads against the reference genome (*M*. *oryzae* strain 70–15; http://www.broadinstitute.org). The minimum and maximum intron lengths were adjusted as five and 2,120 bp based on the exon information of *M*. *oryzae*. The other parameters were set as default values. The Cufflinks (ver. 2.1.1) tool calculated FPKM (fragments per kilobase of transcript per million mapped reads) values using only uniquely mapped reads. A false discovery rate (FDR, *P* < 0.05) was applied for correction. Fold changes were calculated using a modified function, ([FPKM_HYPOXIA_ +1] / [FPKM_NORMOXIA_ +1]).

### Gene ontology

Weight algorithm with Fisher’s exact tests implemented in the R package ‘topGO’ version 2.12.0 was employed to calculate GO term significance for an enrichment analysis of gene sets and visualization of a GO hierarchical structure [25]. GO terms for genes of *M*. *oryzae* were retrieved from Comparative Fungal Genomics Platform (http://cfgp.snu.ac.kr) [26].

### Fungal transformation

Transformation was performed as described previously [27]. Briefly, the *MoADH1* and *MoSRE1* genes were replaced with the constructs having a hygromycin B phosphotranferase gene cassette (*HPH*) with flanking regions of two genes [28]. The primers used for the PCR amplification are listed in S1 Table. After PEG-mediated transformation, mutant candidates were chosen on the selective media (20% sucrose, 1% glucose, 0.3% yeast extract, 0.3% casamino acids, 0.8% agar and 200 ppm of hygromycin). The candidates for *MoADH1* and *MoSRE1* mutants were confirmed by PCR and Southern blot analysis. For genetic complementation of the targeted gene deletion mutant, the fragment containing native promoter region and the full-length ORF of the *MoSRE1* gene was amplified by PCR and used to transform the Δ*Mosre1* mutant with a geneticin resistance gene cassette originated from pII99 [29]. All strains used in this study have been deposited in the Center for Fungal Genetic Resources (http://genebank.snu.ac.kr).

### Southern hybridization assay

Genomic DNA for Southern blotting was prepared by the ‘quick & safe’ method [30] or standard protocols [31]. Restriction enzyme digestion, gel electrophoresis, and Southern hybridization were performed by standard procedures [32]. DNA probes for hybridization were labeled with ^32^P by using the Rediprime II Random Prime Labeling System kit following the manufacturer’s protocol (Amersham Pharmacia Biotech, NJ, USA).

### Assays for fungal development

Growth rate was estimated by measuring diameter of mycelia on the modified complete medium (CM; 0.2% peptone, 1% glucose, 1% casamino acid, 0.1% yeast extract, 0.15% KH_2_PO_4_, 0.05% KCl, 0.6% NaNO_3_, 0.05% MgSO_4_, 0.1% trace element (v/v) and 0.1% vitamin supplement (v/v), pH 6.5) and minimal medium (0.15% KH_2_PO_4_, 0.05% KCl, 0.6% NaNO_3_, 1% glucose, 0.05% MgSO_4_, 0.1% trace element and 0.1% vitamin supplement, pH 6.5) as previously described [33]. The number of asexual spores (conidia) was counted with a hemocytometer under a microscope. Observation of conidiophore development was performed on oatmeal agar media (5% oatmeal (w/v) and 2.5% agar (w/v)) at 16 hours after incubation under a microscope [34]. Conidia were harvested with sterilized distilled water from V8 juice agar media at seven days after incubation and filtered with double-layered Miracloth (CalBiochem, CA, USA). To measure frequencies of germination and appressorium formation, conidial suspensions adjusted to 2 × 10^4^ conidia/ml were spotted onto hydrophobic coverslips with three replicates and incubated in a humid container. Germination of conidia was estimated by the observation of ~100 conidia at four hours after incubation. Appressorium formation rate was determined by the number of formed appressoria to the total number of germinated spores at 16 hours after incubation.

### Pathogenicity and sheath infiltration assays

To evaluate pathogenicity on rice, 10 ml of conidial suspension (10^5^ conidia/ml) were prepared with sterilized distilled water supplemented with Tween 20 (250 ppm final concentration) and sprayed onto rice seedlings (*Oryza sativa* cv. Nakdongbyeo) of three- to four-leaf stage. Rice seedlings were stored in a dew chamber for 24 hours in the dark and then moved into a growth chamber at 25°C, 80% humidity with a photoperiod of 16 hours of light [35]. To monitor lesion development, the pathogenicity assay was also examined by using drop inoculation of conidial suspensions. The 30 μl of conidia suspension adjusted to 10^5^ conidia/ml were dropped onto detached rice leaves and incubated in humid box at room temperature. Rice leaf sheaths were used to observe invasive growth in the rice cells. Conidial suspensions adjusted to 2 × 10^4^ conidia/ml were syringed into the excised sheath cells and incubated in a humid chamber at 25°C [36]. The layers of epidermal cells were observed under the microscope after clipping out chlorophyll-enriched area at 48 hours after inoculation.

### Extracellular oxidative stress sensitivity test

To determine the sensitivity to extracellular oxidative stress, mycelial growth rates were measured on solid CM supplemented H_2_O_2_ (2.5 and 5 mM), 3 mM methyl viologen (paraquat, Aldrich, 856177) and 200 ppm Congo Red (Sigma, C6277). This experiment was performed with three biological replicates.

### Real-time quantitative reverse transcription PCR (qRT-PCR)

cDNA was prepared using ImProm-II Reverse Transcription System (Promega, Madison, WI, USA) according to manufacturer’s recommendation. qRT-PCR reactions were carried out in the mixture of cDNA template (25 ng/ 2 μl), reverse and forward primers(100 nM each in total 3 μl) and Power SYBR Green PCR Master Mix (5 μl, Applied Biosystems, Foster City, CA, USA). The AB7500 Real-Time PCR system was used for amplification and detection (Applied Biosystems, Foster City, CA, USA). PCR condition consists of 40 cycles of 15 s at 95°C, 30 s at 60°C, and 30 s at 72°C after initial denaturation.

## Results and Discussion

### Mapping and quantifying fungal transcriptomes under hypoxia

**Fig 2:**
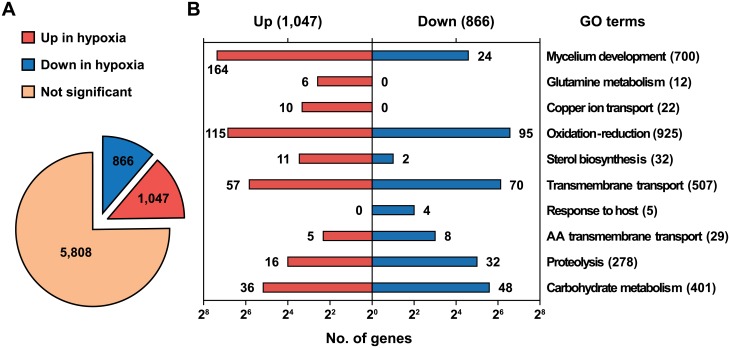
Transcriptional response of *M*. *oryzae* to hypoxic conditions. (A) Expression of annotated transcripts in hypoxia. A total of 7,720 transcripts were selected by the FDR correction (*P* < 0.05). A twofold threshold was applied for significantly regulated genes. (B) Enrichment of specific gene ontology terms under hypoxia. Numbers represent the counts of transcripts supporting the GO terms.

To examine transcriptomic changes of the rice blast fungus in response to hypoxia, we incubated each of 10 plates separately under hypoxia (1% O_2_) and normoxia (20.9% O_2_) for 12 hours after pre-culture on complete medium (CM) for three days. Those 10 plates were used as biological replicates for each conditions. Total RNAs sampled from both conditions were pooled (one lane each) and sequenced using next-generation sequencing technology with three technical repeats (multiplexing with fluorescence dyes; e.g. Hypoxia_0, Hypoxia_1 and Hypoxia_2 in S2 Table). In total, 139 and 167 million reads were generated from hypoxia and normoxia samples, respectively (S2 Table). The original sequencing datasets have been deposited to a public data repository (http://www.ncbi.nlm.nih.gov/geo, accession number: GSE51597). We selected 113 and 140 million reads from hypoxia and normoxia, respectively, as valuable sequences after in-house filtering processes (S2 Table). The TopHat software [24] was employed for large-scale mapping of the filtered reads. Seventy six percent of the filtered reads were mapped onto the genome of *M*. *oryzae* (http://www.broadinstitute.org) with 87% coverage of all transcripts (11,293 out of 12,991, S3 Table). Expression levels of the mapped transcripts were estimated using FPKM (fragments per kilobase of exon per million fragments mapped), which was calculated by the Cufflink software [37]. After applying FDR corrections (*P* < 0.05), we selected a total of 7,720 transcripts (Fig 2A and S3 Table), of which 1,047 transcripts were significantly up-regulated and 866 transcripts were down-regulated in response to hypoxia (two-fold, Fig 2A and S3 Table).

### Genome-wide transcriptomic analysis for hypoxia-responsive genes

**Fig 3:**
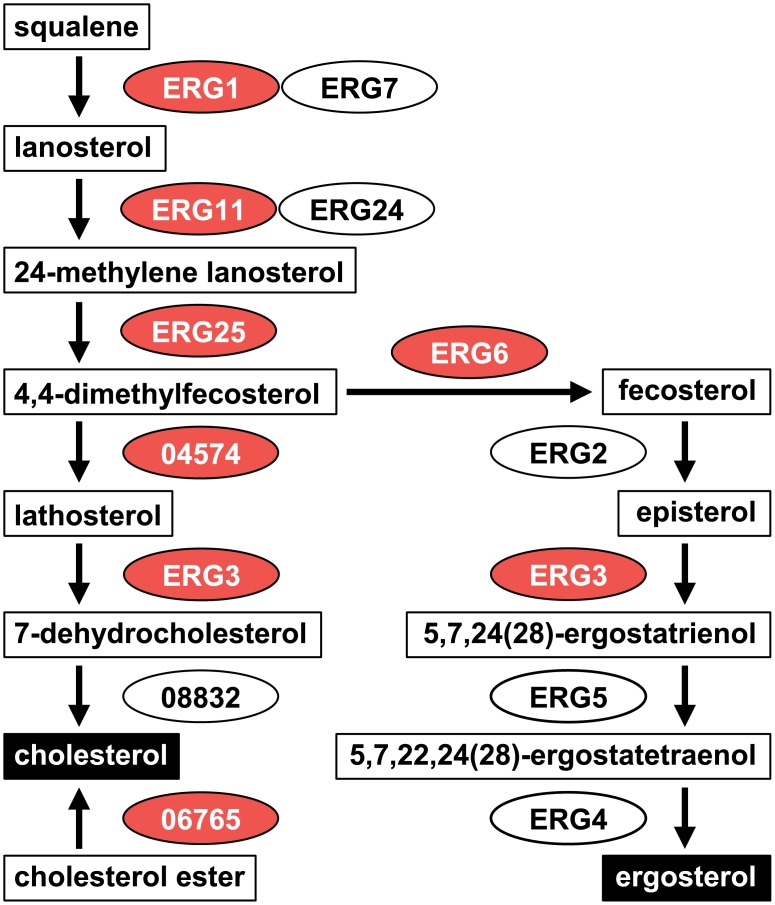
The steroid biosynthesis pathway in *M*. *oryzae*. Intermediates are boxed in white and final products are boxed in black. Genes involved in the pathway are circled. Numbers in the gene names are gene IDs that start with ‘MGG.’ Up-regulated genes are colored in red and down-regulated genes in blue. ‘No significant regulation’ is colored in white.

Gene Ontology (GO) enrichment analysis was conducted to understand functions of genes influenced by hypoxia. The R package ‘topGO’ was used to identify GO terms enriched in the differentially expressed gene sets, giving 16 and 12 enriched GO terms in the up- and down-regulated gene sets, respectively (*P* < 0.05, Fisher's exact test, Table 1 and Fig 2B). The up-regulated genes contained terms involved in mycelial growth: mycelium development (GO:0043581), energy metabolism (GO:0006123 and GO:0015671), and biosynthesis of amino acids (GO:0006541, GO:0009082, GO:0009084, GO:0009450, and GO:0042026), in addition to lipid (GO:0016126 and GO:0072330), phosphate (GO:0070409), and NAD (GO:0034354). As summarized in Fig 1, ‘mycelium development,’ ‘sterol biosynthesis’, and ‘oxygen transport’ terms are closely related in the sterol biosynthesis process. We also examined the steroid biosynthesis pathway in the KEGG database (http://www.genome.jp/kegg-bin/show_pathway?mgr00100). Most genes in this pathway were highly induced under the hypoxic condition and identified as having the ‘sterol biosynthesis’ or ‘oxidation-reduction process’ terms (Fig 3). In the term ‘oxidation-reduction process’, oxygen-consuming enzymes like catalase (MGG_06442) and laccases (MGG_08523 and MGG_11608) were up-regulated, suggesting that lack of oxygen seems to activate oxygen-dependent processes to maintain normal levels of growth in the oxygen-limited environment. An increase in transcripts of catalase and laccase indicates that reactive oxygen species (ROS) might have accumulated in the cell under hypoxia. In addition, genes in the ‘copper ion transport’ term (MGG_02774, MGG_02283, MGG_00930, etc.) are involved in responses to ROS as well as copper homeostasis, supporting ROS accumulation.

**Table 1:** Significant GO terms in response to hypoxia.

GO IDs	Terms		GO term counts		
		Annotated	Observed	Expected	Significance
<Up-regulated in hypoxia >					
GO:0043581	mycelium development	700	164	68.9	0.000
GO:0006541	glutamine metabolic process	12	6	1.2	0.000
GO:0006825	copper ion transport	14	6	1.4	0.001
GO:0055114	oxidation-reduction process	925	115	91.1	0.003
GO:0016126	sterol biosynthetic process	14	5	1.4	0.009
GO:0007623	circadian rhythm	2	2	0.2	0.010
GO:0009450	gamma-aminobutyric acid catabolic process	2	2	0.2	0.010
GO:0015671	oxygen transport	2	2	0.2	0.010
GO:0052372	modulation by symbiont of entry into host	2	2	0.2	0.010
GO:0070409	carbamoyl phosphate biosynthetic process	2	2	0.2	0.010
GO:0009082	branched chain family amino acid biosynthetic process	15	5	1.5	0.012
GO:0006123	mitochondrial electron transport, cytochrome c to oxygen	3	2	0.3	0.027
GO:0034354	de novo NAD biosynthetic process from tryptophan	3	2	0.3	0.027
GO:0042026	protein refolding	3	2	0.3	0.027
GO:0072330	monocarboxylic acid biosynthetic process	27	6	2.7	0.044
GO:0009084	glutamine family amino acid biosynthetic process	21	5	2.1	0.049
<Down-regulated in hypoxia >					
GO:0055085	transmembrane transport	507	70	38.7	0.000
GO:0075136	response to host	5	4	0.4	0.000
GO:0055114	oxidation-reduction process	925	95	70.6	0.001
GO:0003333	amino acid transmembrane transport	29	8	2.2	0.001
GO:0005975	carbohydrate metabolic process	401	48	30.6	0.001
GO:0015847	putrescine transport	2	2	0.2	0.006
GO:0015848	spermidine transport	2	2	0.2	0.006
GO:0008643	carbohydrate transport	39	8	3.0	0.008
GO:0015851	nucleobase transport	7	3	0.5	0.012
GO:0006508	proteolysis	278	32	21.2	0.012
GO:0043419	urea catabolic process	3	2	0.2	0.017
GO:0006071	glycerol metabolic process	8	3	0.6	0.019

In the down-regulated genes, the ‘transport’-related terms such as ‘transmembrane transport’, ‘transport of amino acid, carbohydrates, nucleobase, spermidine, and putrescine’, were enriched, indicating that intra- and extracellular transport was inactive or delayed due to low oxygen (Table 1). The term ‘oxidation-reduction process’ was enriched in the down-regulated genes as well as up-regulated genes, suggesting that the genes belonging to the term might play pivotal roles in adaptation to different oxygen concentrations (Fig 2B). In this term, alcohol oxidases (MGG_ 09072 and MGG_02127) and alcohol dehydrogenases (MGG_04556 and MGG_05519) were down-regulated. Those enzymes participate in the conversion of alcohol to aldehyde using oxygen. Thus, oxygen availability in this fungus would be improved by decreasing expression of those genes. The ‘response to host’ term consists of five genes and four of them (MGG_02863, MGG_05871, MGG_12476, and MGG_10315 (*MPG1*)) were found in the down-regulated transcripts under the hypoxic condition. MPG1 is known as a hydrophobin that functions as a surface sensor when infection-related morphological development begins [38]. Therefore, appressorium formation could be suppressed with the reduced expression of *MPG1*. Given that the genes belonging to the ‘mycelium development’ term were induced under hypoxia, decrease in the *MPG1* transcripts suggests that fungal cells under hypoxia might abandon energy-consuming morphological development and expand the mycelium by simple radial growth to save energy and increase the chance of oxygen uptake. Taken together, the evidence indicated that the rice blast fungus responds to oxygen deprivation mainly by increasing oxygen availability and by shutting down energy-consuming processes such as appressorium formation.

### Comparative analysis of hypoxia-responsive pathways in fungi

We investigated the literature on the genome-wide transcriptional analyses of hypoxia-responsive genes in other fungi. Several microarray datasets whereby wild-type strains were exposed to hypoxia had been generated for *Candida albicans* [39], *Cryptococcus neoformans* [11, 12], *Aspergillus nidulans* [40], *A*. *fumigatus* [41], and *Blastocladiella emersonii* [42]. Conditions used for hypoxia were varied in the studies, including oxygen concentrations (0–1%), composition of other gases (N_2_ and CO_2_), incubation times (1–6 h), and the culture media. However, genes encoding ergosterol biosynthesis, glycolysis, fermentation, fatty acid metabolism, stress response, and hyphal growth were commonly identified in all experiments with different fungi, showing consistency with our GO enrichment results. In addition, we carried out genome-wide comparison of our data with those in *C*. *albicans* [39] and *C*. *neoformans* [11], where 111 and 178 genes were significantly up-regulated under hypoxic conditions, respectively. We identified that 95 and 137 homologous genes of *C*. *albicans* and *C*. *neoformans* exist in the *M*. *oryzae* genome, respectively (BLASTP, e-value cutoff < e^-10^). Among them, 42 (45%) and 37 (25%) were included in the up-regulated genes of *M*. *oryzae* under hypoxia, respectively (S4 Table). Eight genes were commonly induced in all three fungi; these encoded C4-methyl sterol oxidase (ERG25), C5 sterol desaturase (ERG3), fatty acid hydroxylase (SCS7), delta-9 fatty acid desaturase, alcohol dehydrogenase (ADH1), mitochondrial hypoxia responsive domain-containing protein, carboxypeptidase Y, and heat shock protein 70. Considering the total gene numbers of the three fungi, the probability that this happened by chance is extremely low (*P* = 1.4 × e^-11^). Interestingly, the three genes, *ERG25*, *ERG3*, and *SCS7*, had also been identified as SREBP-dependent proteins in *Schizosaccharomyces pombe* [10]. In addition to SREBP (MGG_11534), SREBP cleavage activating protein (SCAP, MGG_08135) and Insig (MGG_06428) are known as regulators for SREBP function and they were highly up-regulated by 5.2- and 3.9-fold in our expression data, respectively. Thus, SREBP-mediated transcriptional responses to hypoxia are well-conserved among these fungi.

We further compared our data to *in planta* transcriptomes which had been analyzed with biotrophic phases of *M*. *oryzae* [43, 44]. The hypoxia-induced genes shared 25 genes (11.7%) with the *in planta* transcriptome (samples harvested at 24 hpi, 213 up-regulated genes in total) and 5 genes (11.1%) in the *in planta* transcriptome (at 36 hpi, 45 genes), respectively (Table 2). This comparative analysis revealed that about ten percent of the *in planta* expressed genes overlapped with the hypoxia-induced genes. And they included genes encoding oxidoreductase, minor extracellular protease, isotrichodermin C-15 hydroxylase which is one of cytochrome P450 proteins (CYPs), laccase, alpha-amylase, amino acid transport system protein, and integral membrane protein. This result suggests that the hypoxia would be one of the environments that the rice blast fungus encountered during infection.

**Table 2:** The *in planta* expressed genes in the hypoxia-induced transcriptome.

Genes	Protein names	Fold change in hypoxia	References
MGG_03374	beta-1,6-galactanase	4.1	[43]
MGG_03671	hypothetical protein	2.1	[43]
MGG_04015	mannan endo-1,6-alpha-mannosidase DCW1	3.3	[43]
MGG_07709	hypothetical protein	2.5	[43]
MGG_10710	oxidoreductase	11.8	[43]
MGG_00050	hypothetical protein	2.3	[44]
MGG_00099	hypothetical protein	3.7	[44]
MGG_00703	MAS3 protein	2.6	[44]
MGG_00715	glucose-repressible protein	4.4	[44]
MGG_01094	hypothetical protein	3.2	[44]
MGG_02329	isotrichodermin C-15 hydroxylase	2.0	[44]
MGG_03988	hypothetical protein	2.5	[44]
MGG_04378	integral membrane protein	5.9	[44]
MGG_04994	plasma membrane H^+^-ATPase	2.7	[44]
MGG_05164	hypothetical protein	2.6	[44]
MGG_05638	hypothetical protein	8.3	[44]
MGG_06542	N amino acid transport system protein	8.3	[44]
MGG_06578	hypothetical protein	3.1	[44]
MGG_07200	plasma membrane ATPase	3.7	[44]
MGG_07341	hypothetical protein	2.4	[44]
MGG_08158	hypothetical protein	2.6	[44]
MGG_08523	laccase-1	31.8	[44]
MGG_08535	hypothetical protein	11.9	[44]
MGG_08811	hypothetical protein	2.3	[44]
MGG_09321	hypothetical protein	6.0	[44]
MGG_09640	alpha-amylase 1	3.6	[44]
MGG_09763	hypothetical protein	2.5	[44]
MGG_12247	hypothetical protein	7.2	[44]
MGG_12655	hypothetical protein	6.1	[44]
MGG_13063	hypothetical protein	3.8	[44]
Sum	30		

### Growth of null mutants for hypoxia-responsive genes

**Fig 4:**
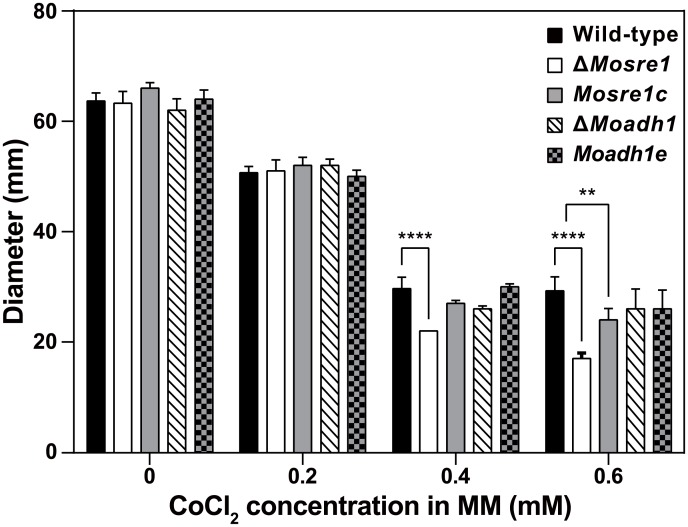
Mycelial growth of *M*. *oryzae* on CoCl_2_-containing medium. Wild-type and two deletion mutants were inoculated on MM with or without CoCl_2_. Plates were incubated at room temperature for 9 days. MM: minimal medium. Two-way ANOVA was performed with Dunnett’s multiple comparison.

We chose two hypoxia-responsive genes—SREBP (*MoSRE1*, MGG_11534) and alcohol dehydrogenase (*MoADH1*, MGG_03880) for further characterization. They were up-regulated in our RNA-seq data (2.6- and 4.1-fold, respectively); up-regulation was confirmed by qRT-PCR. For those genes, deletion mutants were generated by gene replacement via homologous recombination. Correct integration of deletion constructs into the *M*. *oryzae* genome was demonstrated by PCR and Southern hybridization (S1 Fig). We investigated whether those genes are required for overcoming conditions of limited oxygen. As CoCl_2_ (cobalt(II) chloride) has been reported as a chemical hypoxia-mimicking agent in eukaryotic cells [45], the wild-type, mutants and their complementary strains were grown on minimal media (MM) with or without CoCl_2_ (Sigma-Aldrich, 255599). Neither the wild-type nor any of the mutant strains showed any significant differences in mycelial growth on MM (Fig 4). When grown on MM containing CoCl_2_ (0.2 mM to 0.6 mM final concentration), growth of the Δ*Mosre1* mutant was barely detectable at 0.4 mM and 0.6 mM of CoCl_2_, compared to the wild-type and Δ*Moadh1* (Fig 4). That is, growth of the Δ*Mosre1* mutant was more sensitive to hypoxia-mimicking conditions than the others, although there were no differences in radial growth between this mutant and the wild-type when grown on 0.2 mM CoCl_2_-supplemented MM. Interestingly, the *M*. *oryzae* wild-type strain was more sensitive to CoCl_2_ than the other fungi. In *C*. *neoformans* [12] and *A*. *fumigatus* [46], the mutants in which *MoSRE1*-homologous genes had been deleted could not grow on the medium containing 0.6 mM CoCl_2_, while the wild-type strains of both species exhibited normal growth on this medium. On the other hand, the Δ*Mosre1* mutant exhibited a significant growth delay on complete medium (CM) without CoCl_2_ (Table 3). When treated with 0.6 mM, growth of the mutant was unaffected by the addition of CoCl_2_, while the wild-type and complementary strains showed a similar growth defect. Thus, deletion of *MoSRE1* in the wild-type results in a similar effect on fungal growth, as the addition of 0.6 mM CoCl_2_ disturbs normal mycelial growth (mimicry of hypoxia). This observation is also consistent with the suggested role of CoCl_2_ in directly disturbing several oxygen-requiring steps in ergosterol biosynthesis [45]. Taken together, these data suggest that the SREBP-mediated pathway plays a key role in ergosterol biosynthesis, which correlates with hyphal growth in both plant and human pathogens.

**Table 3:** Developmental phenotypes of the transformants for *MoADH1* and *MoSRE1* genes in *M*. *oryzae*.

Strain	Mycelial growth (mm)		Conidiation (x10^4^/ml)	Germination (%)*	Appressorium formation (%)**
	CM	CM+CoCl_2_			
Wild-type	62.0±1.0^A^ ***	39.7±0.6^A^	33.6±4.7^A^	95.7±2.3^A^	93.0±1.7^A^
Δ*Moadh1*	59.3±2.1^A^	42.0±2.6^A^	33.4±4.6^A^	95.0±2.6^A^	94.7±2.1^A^
*Moadh1e*	61.0±2.0^A^	40.7±3.8^A^	32.1±2.7^A^	94.3±1.5^A^	94.0±1.0^A^
Δ*Mosre1*	43.3±4.7^B^	40.7±2.5^A^	81.1±6.7^B^	94.0±2.6^A^	94.7±2.9^A^
*Mosre1c*	61.3±0.6^A^	39.7±0.6^A^	49.9±9.5^C^	94.7±1.2^A^	94.0±0.0^A^

*Germination rate was observed four hours after incubation.

**Appressorium formation rate was observed 16 hours after incubation.

***Two-way ANOVA was performed with Dunnett’s multiple comparison.

### Roles of *MoSRE1* and *MoADH1* in fungal development

**Fig 5:**
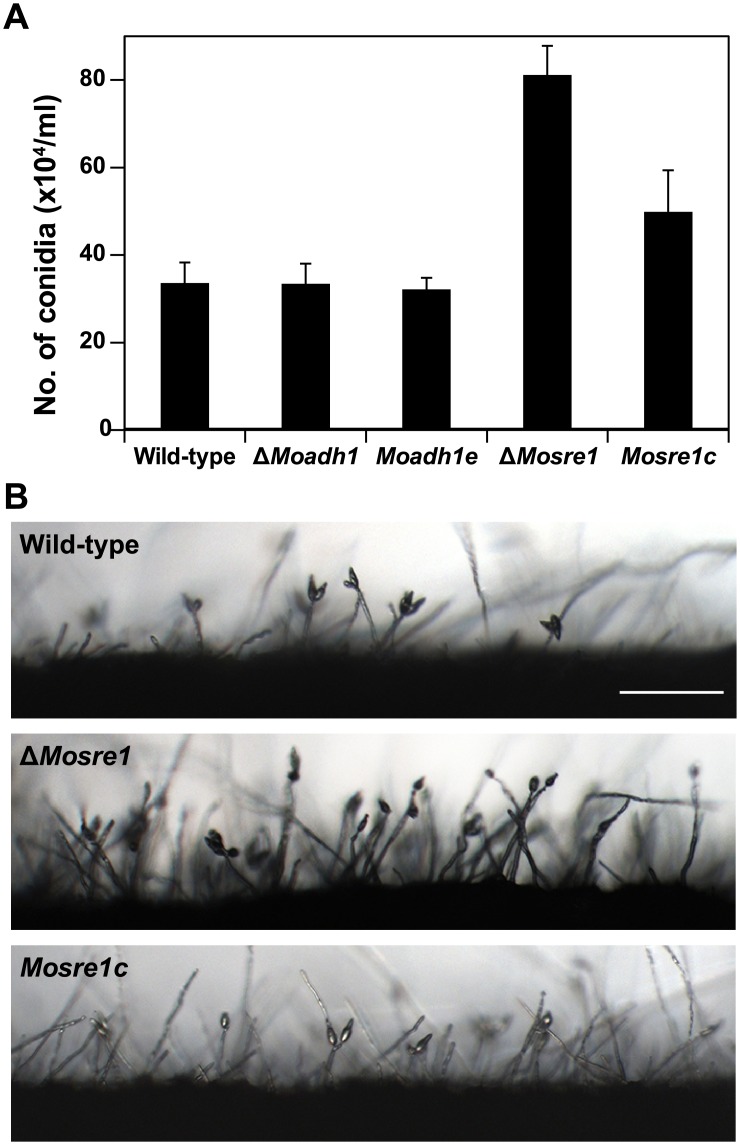
Conidiation of the wild-type, Δ*Moadh1* and Δ*Mosre1*. (A) Conidia were collected from 7-day-old cultures on V8 juice agar plates with 5-ml water. The number of conidia was counted using a hemocytometer under the microscope. (B) Conidiogenesis was monitored under a microscope 16 hours after incubation. Strains were grown on oatmeal agar plates and scraped for inducing conidiogenesis at the same time. Scale bar indicates 100 μm.

To further investigate the roles of the *MoSRE1* and *MoADH1* genes in fungal development and pathogenicity of *M*. *oryzae*, we tested mutants for phenotypes such as conidiation, conidial germination, and appressorium formation. No significant difference was observed in conidial germination (4 h) and appressorium formation (16 h) between the wild-type and either of the two deletion mutants (Table 3). Interestingly, the Δ*Mosre1* mutant produced twice as many conidia than the other strains (Fig 5A and Table 3, *P* < 0.05). Densely developed conidiophores were observed in the Δ*Mosre1* mutant compared to the wild-type and the complemented strain, *Mosre1c* (Fig 5B), suggesting that MoSRE1 has a negative function in conidiation. Because aeration due to unsealing plates generally causes initiation of conidiation in *M*. *oryzae*, the increased conidia in the Δ*Mosre1* mutant was contrary to our expectations. We expected that the oxygen concentration would be decreased in the sealed plates and would stimulate expression of *MoSRE1* as shown in the RNA-seq data. Alternatively, null mutation of *MoSRE1* might promote conidia production in an independent manner. The hypoxia effect via deletion of the *MoSRE1* gene (discussed in Fig 4) could be recognized as a signal that the fungus encountered in an unfavorable environment. It then produced more spores to increase its survival rate under this adverse condition. In addition, the GO enrichment analysis revealed the circadian rhythm term (GO:0007623) was enriched in up-regulated genes (Table 1). The *MoFRQ1* gene (MGG_17344) belongs to this term and its homolog in *Neurospora crassa* has been known as a circadian clock regulator, showing variation in conidiation [47]. In *M*. *oryzae*, the *MoFRQ1* mutant exhibited short conidiophores with dense conidia [48].

### Roles of *MoSRE1* and *MoADH1* in fungal pathogenicity

**Fig 6:**
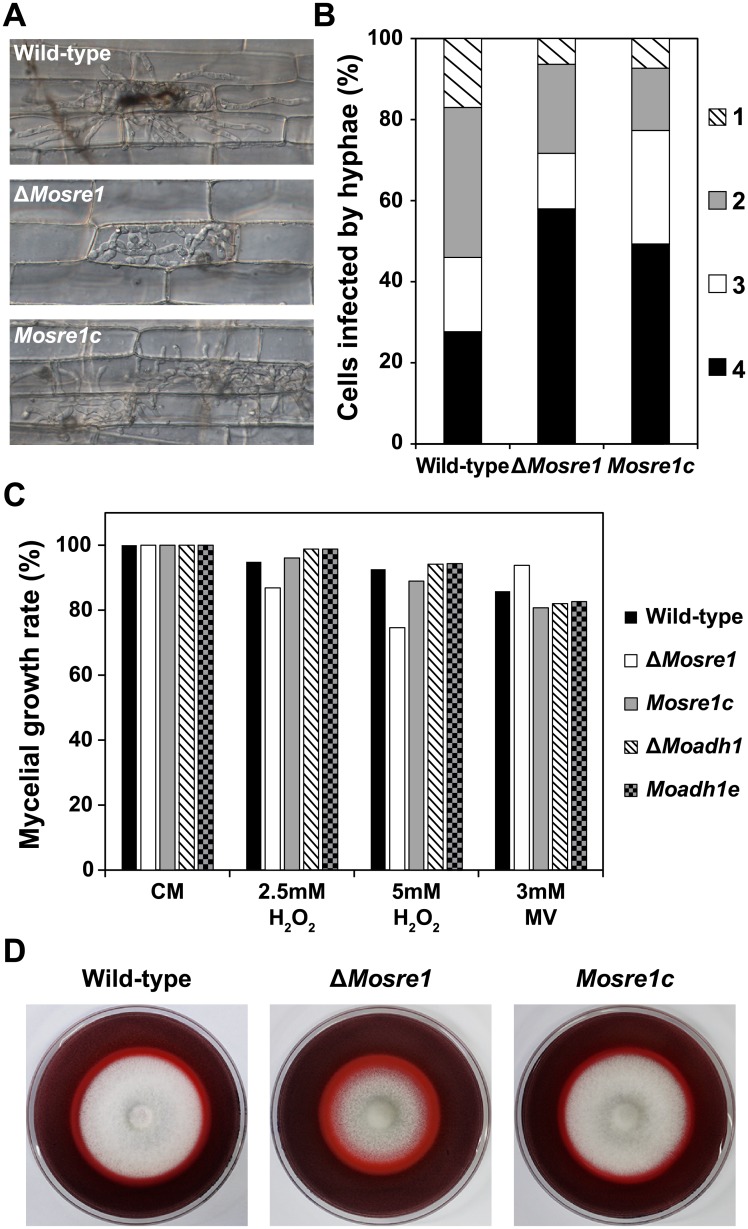
Invasive growth, oxidative stress sensitivity and enzyme activity of the wild-type, Δ*Moadh1* and Δ*Mosre1*. (A) Infectious growth was observed in rice sheath cells. A conidial suspension (2 × 10^4^ conidia/ml) was inoculated into the excised rice sheath. Photographs were taken 48 hours after incubation. Scale bar indicates 20 μm. (B) Frequency of infected rice cells was determined by counting at least 100 appressorium-mediated penetration pegs with three replicates. Invasive growth was observed as described above. 1, Move to adjacent cell; 2, One cell filled; 3, Primary hyphae; 4, No penetration. (C) Extracellular oxidative stress sensitivity of the wild-type and two deletion mutants were examined. Wild-type and two deletion mutants were inoculated on CM and CM including 2.5 or 5 mM H_2_O_2_ and 3 mM methyl viologen (MV). (D) Wild-type, Δ*Mosre1* and *Mosre1c* were inoculated on CM containing 200 ppm Congo Red. Discoloration (halo) of Congo Red was observed at 9 days after incubation.

When a conidial suspension was injected into the rice sheath cells, infectious hyphae of the wild-type and both deletion mutants extended over the adjacent cells after filling inside of the first-infected cell (Fig 6A and 6B). However, penetration and infectious growth of the Δ*Mosre1* mutant were much delayed than those of the wild-type and other mutants. More than 50% of the Δ*Mosre1* conidia produced only appressorium and no penetration peg was observed in them, while less than 30% of the wild-type conidia were marked as the same category, “No penetration” (Fig 6B). Delay in penetration might be explained by the down-regulated genes belonging to the ‘glycerol metabolism’ term (GO:0006071) (Table 1). Because glycerol accumulation increases osmotic pressure within an appressorium [49], a delay in glycerol metabolism led to the penetration defects in the Δ*Mosre1* cells. The proportions of “Primary hyphae”, “One cell filled”, and “Multiple cells” in the mutant were less than those in the wild-type (Fig 6B), supporting overall delay in invasive growth.

In addition, we examined responses to oxidative stresses because hypoxia-induced transcripts such as laccase, catalase, and copper-transport genes might be caused by ROS accumulation in the fungal cell (GO:0055114 and GO:0006825 in Table 1). When the growth on CM was set as 100%, the wild-type showed 95% and 93% of mycelial growth on the CM containing 2.5 mM and 5 mM H_2_O_2_, respectively (Fig 6C). The Δ*Mosre1* mutant showed 87% and 75% of mycelial growth on the media, indicating that the mutant is sensitive to H_2_O_2_. However, the mutant showed opposite trend to the oxidative stress of methyl viologen, a chemical inducer of superoxide free radical. The Δ*Mosre1* mutant was not vulnerable (94%) to methyl viologen while the wild-type and other strains showed slight reduction in response to it (81~86%, Fig 6C). In addition, we tested a degrading activity of Congo Red (a secondary diazo dye) to see extracellular peroxidase or laccase activity of the fungus. Interestingly, the Δ*Mosre1* mutant showed apparent halo compared to the wild-type (Fig 6D). Image analysis revealed that the relative ratio of halo size to mycelial size was higher in the mutant (1.61) than the wild-type (1.40) although total degraded area was almost similar between them (Fig 6D). This indicates that the Δ*Mosre1* mutant generated more extracellular peroxidase or laccase. These results may also explain one of the reasons why the Δ*Mosre1* mutant showed the delayed growth in the sheath inoculation assay (Fig 6A), suggesting that fungal SREBP might be involved in recognition and responses of ROS within host cells. Thus, given that the growth of the Δ*Mosre1* mutant was delayed under hypoxia-mimicking conditions using CoCl_2_ (Fig 4), our observation is the first indirect evidence that hypoxia occurs within plant leaves.

**Fig 7:**
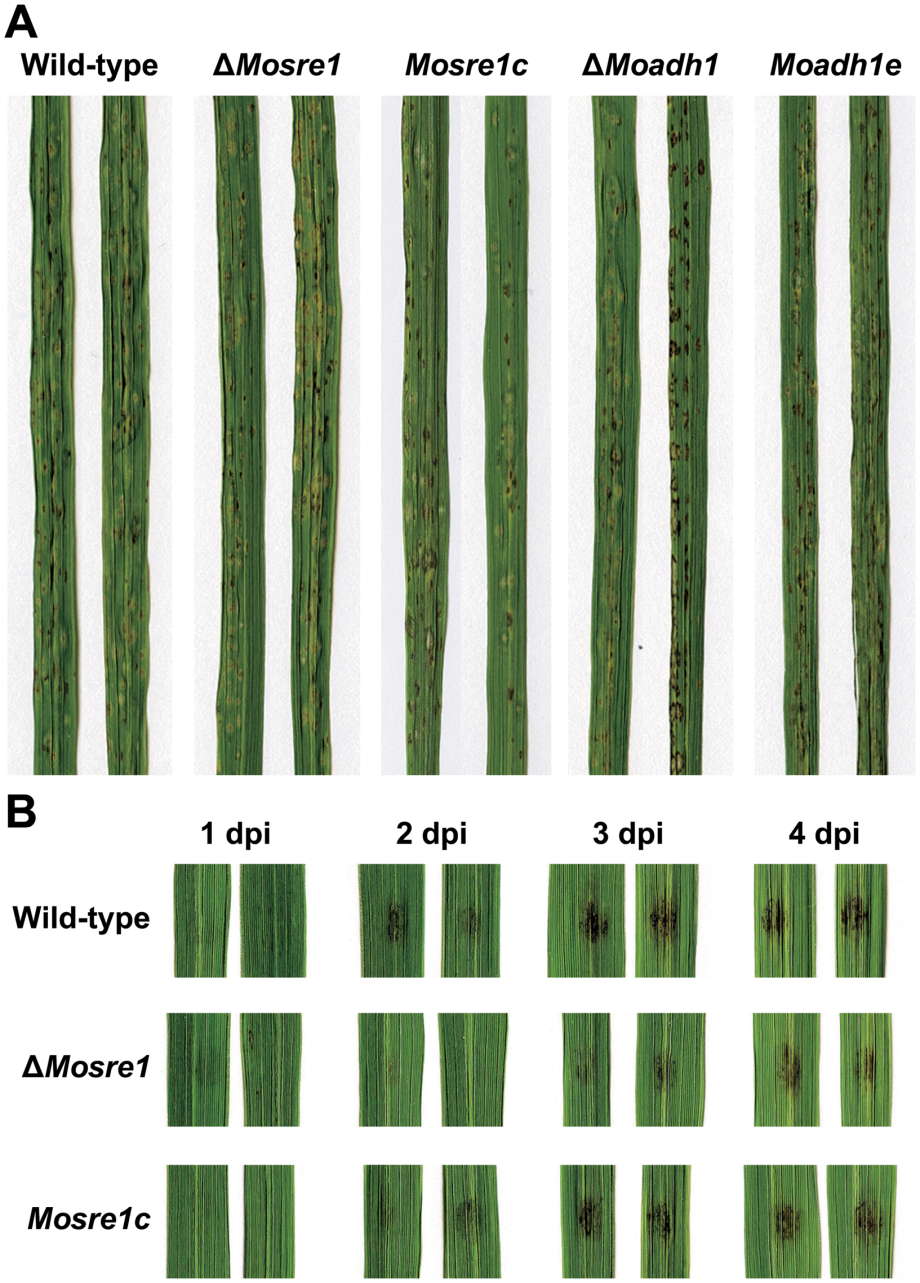
Pathogenicity of wild-type, Δ*Moadh1* and Δ*Mosre1*. (A) The pathogenicity assay was performed by spraying a conidia suspension (5 × 10^4^ conidia/ml) of each strain onto susceptible rice seedlings. Photographs were taken 7 days after inoculation. (B) The pathogenicity assay via drop inoculation was examined. Conidia suspension (10^5^ conidia/ml) was dropped onto detached rice leaves and incubated at room temperature. dpi, days post inoculation.

We then performed spray inoculation assays to determine the effects of the *MoSRE1* and *MoADH1* genes on fungal pathogenicity. Rice leaves were spray-inoculated using a conidial suspension and were observed at seven days post inoculation (dpi). Both mutants exhibited similar disease symptoms compared to the wild-type, *Moadh1e* and *Mosre1c* strains (Fig 7A). To determine when this mutant overcome the delayed growth within host cells, lesion development was monitored using rice leaves by dropping conidial suspensions. Dark lesion areas began to be shown at two days post inoculation with the wild-type conidial suspension while similar patterns of lesions were developed at three days post inoculation in the Δ*Mosre1* mutant (Fig 7B). The delay in Δ*Mosre1* was almost recovered four days post inoculation. These results suggest that Δ*Mosre1* seems to overcome the hypoxic environment in the host cells, which is not the case for SREBP-null mutants of human fungal pathogens within their hosts [13]. It might be explained by the fact that *M*. *oryzae*, unlike human fungal pathogens, switches from a biotrophic to a necrotrophic lifestyle in the middle of the infection.

## Conclusion

Changes in transcriptomes and physiological observations in this study suggest that the hemibiotrophic rice blast fungus might encounter hypoxia in a living host cell during infection. Under hypoxic conditions, this fungus altered transcript levels of diverse sets of genes including those involved in mycelial development, sterol biosynthesis and redox processes. These transcriptional alterations were also observed in human fungal pathogens whose interactions with hosts have been well studied. Especially, ergosterol biosynthesis-related genes (*ERG25*, *ERG3* and *SCS7*) which are associated with the SREBP pathway were induced in *M*. *oryzae* and in all the fungi used for comparison. The SREBP-mediated pathway, in response to a given oxygen concentration, induces ergosterol biosynthesis and hyphal growth in order to scavenge more oxygen in human fungal pathogens. In *M*. *oryzae*, a function for SREBP as an oxygen sensor against hypoxia could be extrapolated from the results that the Δ*Mosre1* mutant exhibited delayed growth in the plant cells and hypoxia-mimicking environment. In addition, SREBP also functions as a negative regulator for conidiation. Our data provides a new platform to decipher molecular machinery underpinning surveillance of hypoxic conditions during interactions between plant hosts and fungal pathogens.

## Supporting Information

S1 Fig Southern blot analyses of the Δ*Moadh1* and Δ*Mosre1* mutants. (A) Δ*Moadh1*. Wild-type and the Δ*Moadh1* mutant had a 1.8-kb and 3.9-kb *Sal*I fragment, respectively. (B) Δ*Mosre1*. Wild-type and the Δ*Mosre1* mutant had a 5.3-kb and 3.5-kb *Xba*I fragment, respectively.
(PPTX)

S1 Table Oligo sequences used in this study. (DOCX)

S2 Table Summary of mapping statistics. (DOCX)

S3 Table Selected transcripts in hypoxia transcriptome. (XLSX)

S4 Table Comparative analysis of hypoxia transcriptomes. (XLSX)

## References

[1] Ernst JF, Tielker D. Responses to hypoxia in fungal pathogens. Cell Microbiol. 2009;11(2):183–90. 10.1111/j.1462-5822.2008.01259.x 19016786

[2] Semenza GL. Life with oxygen. Science. 2007;318(5847):62–4. 17916722 10.1126/science.1147949

[3] Packer L, Fuehr K. Low oxygen concentration extends lifespan of cultured human diploid cells. Nature. 1977;267(5610):423–5. 876356 10.1038/267423a0

[4] Jacobson MD, Raff MC. Programmed cell death and Bcl-2 protection in very low oxygen. Nature. 1995;374(6525):814–6. 7536895 10.1038/374814a0

[5] van Dongen JT, Schurr U, Pfister M, Geigenberger P. Phloem metabolism and function have to cope with low internal oxygen. Plant Physiol. 2003;131(4):1529–43. 12692313 10.1104/pp.102.017202 PMC166912

[6] Zitomer RS, Carrico P, Deckert J. Regulation of hypoxic gene expression in yeast. Kidney Int. 1997;51(2):507–13. 9027731 10.1038/ki.1997.71

[7] Igwe E, Essler S, Al-Furoukh N, Dehne N, Brune B. Hypoxic transcription gene profiles under the modulation of nitric oxide in nuclear run on-microarray and proteomics. BMC Genomics. 2009;10(1):408. 19725949 10.1186/1471-2164-10-408 PMC2743718

[8] Erecinska M, Silver IA. Tissue oxygen tension and brain sensitivity to hypoxia. Respir Physiol. 2001;128(3):263–76. 11718758 10.1016/s0034-5687(01)00306-1

[9] Grahl N, Puttikamonkul S, Macdonald JM, Gamcsik MP, Ngo LY, Hohl TM, et al *In vivo* hypoxia and a fungal alcohol dehydrogenase influence the pathogenesis of invasive pulmonary aspergillosis. PLoS Path. 2011;7(7):e1002145. 10.1371/journal.ppat.1002145 PMC3141044 21811407

[10] Hughes AL, Todd BL, Espenshade PJ. SREBP pathway responds to sterols and functions as an oxygen sensor in fission yeast. Cell. 2005;120(6):831–42. 15797383 10.1016/j.cell.2005.01.012

[11] Chun CD, Liu OW, Madhani HD. A link between virulence and homeostatic responses to hypoxia during infection by the human fungal pathogen *Cryptococcus neoformans* . PLoS Path. 2007;3(2):e22. 10.1371/journal.ppat.0030022 PMC1803011 17319742

[12] Chang YC, Bien CM, Lee H, Espenshade PJ, Kwon-Chung KJ. Sre1p, a regulator of oxygen sensing and sterol homeostasis, is required for virulence in *Cryptococcus neoformans* . Mol Microbiol. 2007;64(3):614–29. 17462012 10.1111/j.1365-2958.2007.05676.x

[13] Willger SD, Puttikamonkul S, Kim KH, Burritt JB, Grahl N, Metzler LJ, et al A sterol-regulatory element binding protein is required for cell polarity, hypoxia adaptation, azole drug resistance, and virulence in *Aspergillus fumigatus* . PLoS Path. 2008;4(11):e1000200. 10.1371/journal.ppat.1000200 PMC2572145 18989462

[14] Geigenberger P. Response of plant metabolism to too little oxygen. Curr Opin Plant Biol. 2003;6(3):247–56. 12753974 10.1016/s1369-5266(03)00038-4

[15] Ober ES, Sharp RE. A microsensor for direct measurement of O_2_ partial pressure within plant tissues. J Exp Bot. 1996;47(296):447–54.

[16] Rolletschek H, Borisjuk L, Koschorreck M, Wobus U, Weber H. Legume embryos develop in a hypoxic environment. J Exp Bot. 2002;53(371):1099–107. 11971921 10.1093/jexbot/53.371.1099

[17] Rijnders JGHM, Armstrong W, Darwent MJ, Blom CWPM, Voesenek LACJ. The role of oxygen in submergence-induced petiole elongation in *Rumex palustris*: *in situ* measurements of oxygen in petioles of intact plants using micro-electrodes. New Phytol. 2000;147(3):497–504. 10.1046/j.1469-8137.2000.00727.x 33862947

[18] Khush G, Jena KK. Current status and future prospects for research on blast resistance in rice (*Oryza sativa* L.) In: Wang G-L, Valent B, editors. Advances in Genetics, Genomics and Control of Rice Blast Disease: Springer; 2009 p. 1–10.

[19] Kankanala P, Czymmek K, Valent B. Roles for rice membrane dynamics and plasmodesmata during biotrophic invasion by the blast fungus. Plant Cell. 2007;19(2):706–24. 17322409 10.1105/tpc.106.046300 PMC1867340

[20] Howard RJ, Valent B. Breaking and entering: Host penetration by the fungal rice blast pathogen *Magnaporthe grisea* . Annu Rev Microbiol. 1996;50:491–512. 8905089 10.1146/annurev.micro.50.1.491

[21] Khang CH, Berruyer R, Giraldo MC, Kankanala P, Park SY, Czymmek K, et al Translocation of *Magnaporthe oryzae* effectors into rice cells and their subsequent cell-to-cell movement. Plant Cell. 2010;22(4):1388–403. 10.1105/tpc.109.069666 20435900 PMC2879738

[22] Schroeder A, Mueller O, Stocker S, Salowsky R, Leiber M, Gassmann M, et al The RIN: an RNA integrity number for assigning integrity values to RNA measurements. BMC Mol Biol. 2006;7:3 16448564 10.1186/1471-2199-7-3 PMC1413964

[23] Patel RK, Jain M. NGS QC toolkit: a toolkit for quality control of next generation sequencing data. PLoS ONE. 2012;7(2):e30619 10.1371/journal.pone.0030619 22312429 PMC3270013

[24] Trapnell C, Pachter L, Salzberg SL. TopHat: discovering splice junctions with RNA-Seq. Bioinformatics. 2009;25(9):1105–11. 10.1093/bioinformatics/btp120 19289445 PMC2672628

[25] Alexa A, Rahnenfuhrer J, Lengauer T. Improved scoring of functional groups from gene expression data by decorrelating GO graph structure. Bioinformatics. 2006;22(13):1600–7. 16606683 10.1093/bioinformatics/btl140

[26] Choi J, Cheong K, Jung K, Jeon J, Lee GW, Kang S, et al CFGP 2.0: a versatile web-based platform for supporting comparative and evolutionary genomics of fungi and Oomycetes. Nucleic Acids Res. 2013;41(D1):D714–D9. 23193288 10.1093/nar/gks1163 PMC3531191

[27] Park J, Kong S, Kim S, Kang S, Lee Y-H. Roles of forkhead-box transcription factors in controlling development, pathogenicity, and stress response in *Magnaporthe oryzae* . Plant Pathol J. 2014;30(2):136–50. 10.5423/PPJ.OA.02.2014.0018 25288996 PMC4174854

[28] Choi J, Kim Y, Kim S, Park J, Lee YH. *MoCRZ1*, a gene encoding a calcineurin-responsive transcription factor, regulates fungal growth and pathogenicity of *Magnaporthe oryzae* . Fungal Genet Biol. 2009;46(3):243–54. 10.1016/j.fgb.2008.11.010 19111943

[29] Yi M, Chi MH, Khang CH, Park SY, Kang S, Valent B, et al The ER chaperone LHS1 is involved in asexual development and rice infection by the blast fungus *Magnaporthe oryzae* . Plant Cell. 2009;21(2):681–95. 10.1105/tpc.107.055988 19252083 PMC2660637

[30] Chi MH, Park SY, Lee YH. A quick and safe method for fungal DNA extraction. Plant Pathol J. 2009;25(1):108–11.

[31] Choi J, Park J, Jeon J, Chi MH, Goh J, Yoo SY, et al Genome-wide analysis of T-DNA integration into the chromosomes of *Magnaporthe oryzae* . Mol Microbiol. 2007;66(2):371–82. 17850257 10.1111/j.1365-2958.2007.05918.x PMC2169514

[32] Sambrook J, Russel DW. Molecular Cloning: A Laboratory Manual. 3rd ed. ed. Cold Spring Harbor, NY, USA: Cold Spring Harbor Laboratory Press; 2001.

[33] Talbot NJ, Ebbole DJ, Hamer JE. Identification and characterization of *MPG1*, a gene involved in pathogenicity from the rice blast fungus *Magnaporthe grisea* . Plant Cell. 1993;5(11):1575–90. 8312740 10.1105/tpc.5.11.1575 PMC160387

[34] Lau GW, Hamer JE. *Acropetal*: A genetic locus required for conidiophore architecture and pathogenicity in the rice blast fungus. Fungal Genet Biol. 1998;24(1–2):228–39. 9742203 10.1006/fgbi.1998.1053

[35] Valent B, Farrall L, Chumley FG. *Magnaporthe grisea* genes for pathogenicity and virulence identified through a series of backcrosses. Genetics. 1991;127(1):87–101. 2016048 10.1093/genetics/127.1.87 PMC1204315

[36] Koga H, Dohi K, Nakayachi O, Mori M. A novel inoculation method of *Magnaporthe grisea* for cytological observation of the infection process using intact leaf sheaths of rice plants. Physiol Mol Plant Pathol. 2004;64(2):67–72.

[37] Trapnell C, Williams BA, Pertea G, Mortazavi A, Kwan G, van Baren MJ, et al Transcript assembly and quantification by RNA-Seq reveals unannotated transcripts and isoform switching during cell differentiation. Nat Biotechnol. 2010;28(5):511–7. 10.1038/nbt.1621 20436464 PMC3146043

[38] Talbot NJ, Kershaw MJ, Wakley GE, deVries OMH, Wessels JGH, Hamer JE. *MPG1* encodes a fungal hydrophobin involved in surface interactions during infection-related development of *Magnaporthe grisea* . Plant Cell. 1996;8(6):985–99. 12239409 10.1105/tpc.8.6.985 PMC161153

[39] Setiadi ER, Doedt T, Cottier F, Noffz C, Ernst JF. Transcriptional response *Candida albicans* to hypoxia: Linkage of oxygen sensing and Efg1p-regulatory networks. J Mol Biol. 2006;361(3):399–411. 16854431 10.1016/j.jmb.2006.06.040

[40] Masuo S, Terabayashi Y, Shimizu M, Fujii T, Kitazume T, Takaya N. Global gene expression analysis of *Aspergillus nidulans* reveals metabolic shift and transcription suppression under hypoxia. Mol Genet Genomics. 2010;284(6):415–24. 10.1007/s00438-010-0576-x 20878186

[41] Barker BM, Kroll K, Vodisch M, Mazurie A, Kniemeyer O, Cramer RA. Transcriptomic and proteomic analyses of the *Aspergillus fumigatus* hypoxia response using an oxygen-controlled fermenter. BMC Genomics. 2012;13(1):62. 22309491 10.1186/1471-2164-13-62 PMC3293747

[42] Camilo CM, Gomes SL. Transcriptional response to hypoxia in the aquatic fungus *Blastocladiella emersonii* . Eukaryot Cell. 2010;9(6):915–25. 10.1128/EC.00047-10 20418381 PMC2901646

[43] Mosquera G, Giraldo MC, Khang CH, Coughlan S, Valent B. Interaction transcriptome analysis identifies *Magnaporthe oryzae* BAS1-4 as biotrophy-associated secreted proteins in rice blast disease. Plant Cell. 2009;21: 1273–1290. 10.1105/tpc.107.055228 19357089 PMC2685627

[44] Kawahara Y, Oono Y, Kanamori H, Matsumoto T, Itoh T, Minami E. Simultaneous RNA-seq analysis of a mixed transcriptome of rice and blast fungus interaction. PLoS One. 2012;7: e49423 10.1371/journal.pone.0049423 23139845 PMC3490861

[45] Lee H, Bien CM, Hughes AL, Espenshade PJ, Kwon-Chung KJ, Chang YC. Cobalt chloride, a hypoxia-mimicking agent, targets sterol synthesis in the pathogenic fungus *Cryptococcus neoformans* . Mol Microbiol. 2007;65(4):1018–33. 17645443 10.1111/j.1365-2958.2007.05844.x

[46] Blatzer M, Barker BM, Willger SD, Beckmann N, Blosser SJ, Cornish EJ, et al SREBP coordinates iron and ergosterol homeostasis to mediate triazole drug and hypoxia responses in the human fungal pathogen *Aspergillus fumigatus* . PLoS Genet. 2011;7(12):e1002374 10.1371/journal.pgen.1002374 22144905 PMC3228822

[47] McClung CR, Fox BA, Dunlap JC. The *Neurospora* clock gene *frequency* shares a sequence element with the *Drosophila* clock gene *period* . Nature. 1989;339(6225):558–62. 2525233 10.1038/339558a0

[48] Park J, Lee Y-H. Bidirectional-genetics platform, a dual-purpose mutagenesis strategy for filamentous fungi. Eukaryot Cell. 2013;12(11):1547–53. 10.1128/EC.00234-13 24058171 PMC3837931

[49] de Jong JC, McCormack BJ, Smirnoff N, Talbot NJ. Glycerol generates turgor in rice blast. Nature. 1997;389(6648):244–5.

