# Supplementary material for: Genome-Wide Analysis of Hypoxia-Responsive Genes in the Rice Blast Fungus, Magnaporthe oryzae
Source: PLoS One. 2015 Aug 4;10(8):e0134939. doi: 10.1371/journal.pone.0134939 (PMC4524601; doi:10.1371/journal.pone.0134939)
Supplement: S1 Fig — (A) ΔMoadh1. Wild-type and the ΔMoadh1 mutant had a 1.8-kb and 3.9-kb SalI fragment, respectively. (B) ΔMosre1. Wild-type and the ΔMosre1 mutant had a 5.3-kb and 3.5-kb XbaI fragment, respectively. (PPTX) [file pone.0134939.s001.pptx]

## Slide 1
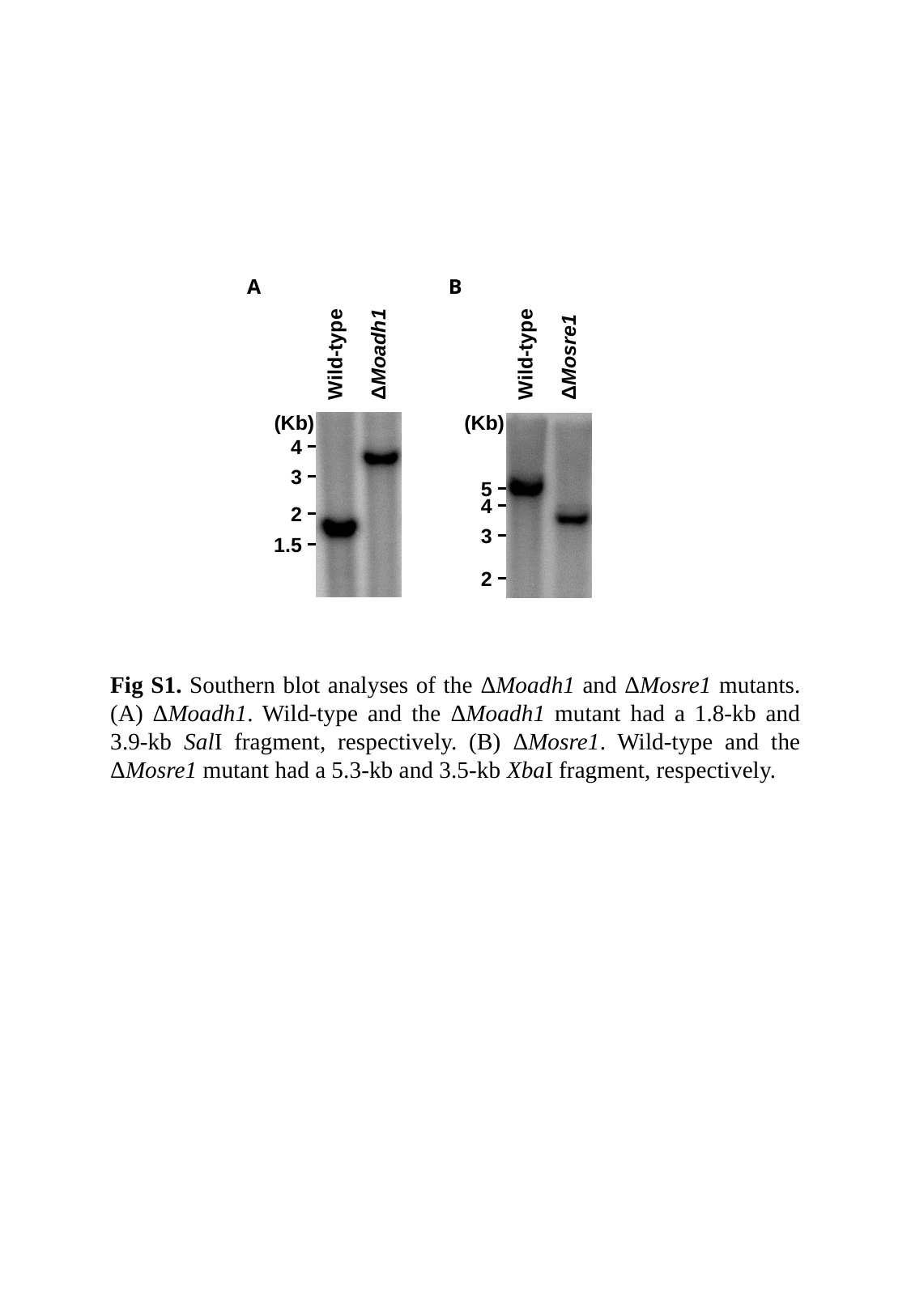

A
B
Wild-type
ΔMoadh1
(Kb)
4
3
2
1.5
Wild-type
ΔMosre1
(Kb)
5
4
3
2
Fig S1. Southern blot analyses of the ΔMoadh1 and ΔMosre1 mutants. (A) ΔMoadh1. Wild-type and the ΔMoadh1 mutant had a 1.8-kb and 3.9-kb SalI fragment, respectively. (B) ΔMosre1. Wild-type and the ΔMosre1 mutant had a 5.3-kb and 3.5-kb XbaI fragment, respectively.
