# Supplementary material for: Genome-Wide Analysis of Hypoxia-Responsive Genes in the Rice Blast Fungus, Magnaporthe oryzae
Source: PLoS One. 2015 Aug 4;10(8):e0134939. doi: 10.1371/journal.pone.0134939 (PMC4524601; doi:10.1371/journal.pone.0134939)
Supplement: S1 Table — (DOCX) [file pone.0134939.s002.docx]

Table S1. Oligo sequences used in this study

| Name | Sequence (5’ → 3’) |
| --- | --- |
| HPH_F | GGCTTGGCTGGAGCTAGTGGAGG |
| HPH_R | GTTGGTGTCGATGTCAGCTCCGGAG |
| MGG_03880_UF | CATGCTTACATTTGGCTGGA |
| MGG_03880_UR | CCTCCACTAGCTCCAGCCAAGCCCGTCGTTCAAATTTGTCTGG |
| MGG_03880_DF | GTTGGTGTCGATGTCAGCTCCGGAGGATTGCTTCCGTATGTCTGG |
| MGG_03880_DR | CAAGGTTTTCCGCCATCTTA |
| MGG_03880_NST_F | GGATCCGGGGTATCAGGTAT |
| MGG_03880_NST_R | TCACGCAGTAGAATCGTTGC |
| MGG_11534_UF | CTCAATTGTTGTGCCCAGTG |
| MGG_11534_UR | CCTCCACTAGCTCCAGCCAAGCCGGGCTAGGAAGTGGACGGTA |
| MGG_11534_DF | GTTGGTGTCGATGTCAGCTCCGGAGCGATCGACTAAGAAGCCAGG |
| MGG_11534_DR | TTGAGTTCATGCGTTGGGTA |
| MGG_11534_NST_F | AGGTGTCTCGGGTTTGTGAC |
| MGG_11534_NST_R | TCGTCATAGTGCTGAGCCAC |
