# Supplementary material for: Genome-Wide Analysis of Hypoxia-Responsive Genes in the Rice Blast Fungus, Magnaporthe oryzae
Source: PLoS One. 2015 Aug 4;10(8):e0134939. doi: 10.1371/journal.pone.0134939 (PMC4524601; doi:10.1371/journal.pone.0134939)
Supplement: S2 Table — (DOCX) [file pone.0134939.s003.docx]

**Table S2.** Summary of mapping statistics

|  | Hypoxia | Normoxia |
| --- | --- | --- |
| Total reads | 139,432,210 | 166,836,634 |
| Filtered reads | 113,885,992 (81.7%) | 140,079,644 (84.0%) |
| Uniquely mapped reads | 85,260,776 (74.9%) | 107,767,588 (76.9%) |
